# Groundwater denitrification using electro-assisted autotrophic processes: exploring bacterial community dynamics in a single-chamber reactor

**DOI:** 10.3389/fbioe.2025.1475589

**Published:** 2025-01-22

**Authors:** Javiera Toledo-Alarcón, Eduardo Ortega-Martinez, Javier Pavez-Jara, Oscar Franchi, Ivan Nancucheo, Héctor Zuñiga-Barra, Jose Luis Campos, David Jeison

**Affiliations:** ^1^ Facultad de Ingeniería y Ciencias, Universidad Adolfo Ibáñez, Viña del Mar, Chile; ^2^ Escuela de Ingeniería Bioquímica, Pontificia Universidad Católica de Valparaíso, Valparaíso, Chile; ^3^ Facultad de Ciencias Naturales, Matemática y del Medio Ambiente, Universidad Tecnológica Metropolitana, Ñuñoa, Chile; ^4^ Facultad de Ingeniería, Arquitectura y Diseño, Universidad San Sebastián, Concepción, Chile

**Keywords:** electroactive bacterial community, desulfosporosinus genus, bioelectrochemical system, autotrophic denitrification, nitrate removal

## Abstract

Nitrate, a major groundwater pollutant from anthropogenic activities, poses serious health risks when present in drinking water. Denitrification using bio-electrochemical reactors (BER) offers an innovative technology, eco-friendly solution for nitrate removal from groundwater. BER use electroactive bacteria to reduce inorganic compounds like nitrate and bicarbonate by transferring electrons directly from the cathode. In our work, two batch BER were implemented at 1V and 2V, using anaerobic digestate from a full-scale wastewater treatment plant as inoculum. Nitrate, nitrite, sulfate, total ammoniacal nitrogen, and 16S rRNA analysis of bacterial community, were monitored during BER operation. The results showed effective nitrate removal in all BERs, with denitrification rate at 1V and 2V higher than the Control system, where endogenous respiration drove the process. At 1V, complete nitrate conversion to N_2_ occurred in 4 days, while at 2V, it took 14 days. The slower rate at 2V was likely due to O_2_ production from water electrolysis, which competed with nitrate as final electron acceptor. Bacterial community analysis confirmed the electroactive bacteria selection like the genus *Desulfosporosinus* and *Leptolinea*, confirming electrons transfer without an electroactive biofilm. Besides, *Hydrogenophaga* was enhanced at 2V likely due to electrolytically produced H_2_. Sulfate was not reduced, and total ammoniacal nitrogen remained constant indicating no dissimilatory nitrite reduction of ammonia. These results provide a significant contribution to the scaling up of electro-assisted autotrophic denitrification and its application in groundwater remediation, utilizing a simple reactor configuration-a single-chamber, membrane-free design- and a conventional power source instead of a potentiostat.

## 1 Introduction

Groundwater is an important fresh water source in the world, supplying about 50% of the water used for domestic purposes and about 25% of all water used for irrigation (UNESCO - World Water Assessment Programme, 2022). However, groundwater quality can be compromised due to diverse anthropogenic activities. For instance, agricultural activities can contaminate groundwater with nitrates (NO_3_^−^), hampering the ability of natural systems to decontaminate themselves (Rezvani et al., 2019; Kurwadkar et al., 2020). Moreover, frequent human consumption of water containing nitrates can be linked to serious health issues including fifteen forms of cancer and two types of birth abnormalities (Alizadeh et al., 2024). In this regard, the World Health Organization (WHO) recommends a maximum nitrate concentration of 50 mg/L in drinking water. Nonetheless, recent evidence suggests that this limit may be too high, and it may increase the risk of suffering prostate and colon cancer, as well as congenital diseases (Schullehner et al., 2018; Damania et al., 2019; Donat-Vargas et al., 2023). For these reasons, removing nitrate from groundwater is critical to ensure a safe drinking water supply for human consumption.

The most used technologies to remove nitrate from groundwater are reverse osmosis and ion exchange resins (Jensen et al., 2014; Abascal et al., 2022). However, physical nitrate removal methods generate streams with nitrate levels up to 10 times more concentrated than in the influent (Belkacem et al., 2007; Archna Sharma and Sobti, 2012; Scholes et al., 2021), which need further purification. In this context, technological interventions to return nitrogen to the geological cycle in an environmentally friendly way are urgently required.

Emerging technologies such as autotrophic denitrification using bioelectrochemical reactors (BER) have arisen as a promising alternative to remove nitrate from groundwater. These technologies can improve groundwater’s quality reaching levels that can be used to meet human consumption standards. During autotrophic denitrification using BER, nitrate is reduced using an electrical current as the sole electron source, and inorganic carbon as a carbon source (Ceballos-Escalera et al., 2024). Among the advantages of using BER are the absence of chemicals addition, no brine generation, and potentially competitive prices compared with similar technologies (Twomey et al., 2010; Cecconet et al., 2018).

During the BER process, nitrate reduction depends on the voltage applied, as well as the interaction between the denitrifying bacterial community with the polarized electrodes (Kondaveeti et al., 2014; Ortega-Martínez et al., 2024). Consequently, a specific electroactive bacterial community is needed that can harvest electrons from the electrode. Few bacteria are known that can directly utilise electrons from a cathode, as they need specific pathways for extracellular electron transfer (EET) and are commonly identified by growing as a biofilm on the polarised electrode surface (Moscoviz et al., 2016; Lovley, 2017).

EET pathways have been well described in the bacterial genus *Geobacter* and *Shewanella oneidensis* (Moscoviz et al., 2016; Lovley, 2017), but new research has revealed the ability of other bacteria such as *Lactiplantibacillus plantarum* and *Clostridium pasteurianum* to harvest electrons from a polarized cathode by mechanisms that are still being further elucidated (Choi et al., 2014; Tejedor-Sanz et al., 2023). Various metabolic pathways are now known to be conducted using electrons directly from the cathode, combined with the reduction of sulfate, nitrate, iron, and CO_2_, which can be carried out by microorganisms from different archaeal and bacterial phyla such as *Euryarchaeota, Actinobacteria, Firmicutes* and *Proteobacteria* (deCamposRodrigues and Rosenbaum, 2014; Logan et al., 2019).

In full-scale applications, nitrate will probably not be the sole electron acceptor that the microorganisms can use to harvest energy. Commonly nitrate is accompanied by other potentially reduceable species like sulfates. Sulfate in groundwater originates from different sources, such as volcanoes, oxidation of igneous sulfides and organic matter, fertilizers, and detergents (Clark and Fritz, 2013; Jorquera et al., 2015; Novak et al., 2021). During autotrophic denitrification, sulfate and nitrate in groundwater compete as the final electron acceptor during cell growth. However, this competition is determined by the thermodynamics of the metabolic reactions involved. Equation 1 and Equation 2 show the catabolic reduction reaction of nitrate and sulfate under standard conditions, respectively (Dolfing and Hubert, 2017). As can be seen from these equations, in almost all cases bacteria will preferentially reduce nitrate over sulfate, as they can harvest more energy per electron of each molecule (more negative $∆G^{0}\prime$ ). However, thermodynamic analysis of catabolic reactions does not determine whether bacteria have the necessary biochemical machinery to conduct these reactions or the rate at which they occur. These are determined by the species in the bacterial community and the environmental conditions under which the biochemical conversions are conducted.

$$\frac{1}{5}{\text{NO}}_{3}^{‐}+\frac{6}{5}H^{+}+e^{‐}↔\frac{1}{10}N_{2}+\frac{3}{5}H_{2}O∆G^{0}\prime =‐112.1\frac{\text{kJ}}{e^{‐}}$$ (1)

$$\frac{1}{8}{\text{SO}}_{4}^{2‐}+\frac{9}{8}H^{+}+e^{‐}↔\frac{1}{8}{\text{HS}}^{‐}+\frac{1}{2}H_{2}O∆G^{0}\prime =‐19\frac{\text{kJ}}{e^{‐}}$$ (2)

This paper aims to evaluate the bacterial community dynamics during nitrate reduction of a sulfate-rich synthetic groundwater in a single-chamber BER. The working potential differences were set at 1V and 2V, to provide the bacteria with two possible denitrification mechanisms depending on the electron source: (i) H_2_ produced by electrolysis of water at 2V, and (ii) direct electron consumption at 1V. The novelty of our research, compared to previous similar efforts, is the use of a power supply instead of a potentiostat to establish the working potential differences, as well as the use of a membrane-free BER. This allows progress towards the BER application on an industrial scale through the implementation of a simpler system.

## 2 Materials and methods

### 2.1 BER configuration and start-up

BER comprised two batch, completely stirred reactors with a working volume of 600 mL, equipped with 2 carbon plate electrodes fixed at 6 cm, and a surface of 10 cm^2^. Two electric potential differences were tested and compared: 1V and 2V, which were set using a power supply model ODP3033 (OWON Technology Inc., China). The reactors were continuously stirred at 300 RPM using a magnetic stirrer and operated at room temperature without pH control. The reactors’ headspace were flushed with dinitrogen for 5 min to remove oxygen and promote anoxic conditions, and sealed to prevent air intrusion. A third reactor without electrodes was used as a control (named Control) and all experiments were performed in duplicate. In addition, abiotic controls without micro-organisms were carried out at 1V and 2V.

### 2.2 Inoculum and groundwater medium

Digestate from the wastewater treatment plant (WWTP) La Farfana (Maipú, Chile) was used as inoculum. The WWTP contains eight 15,000 m^3^ completely stirred anaerobic reactors which digest a mix of primary and secondary sludge, with a hydraulic retention time of 16–19 days. Once sampled, the inoculum was resuspended in a synthetic medium to reach a concentration of 1.5 gVSS/L (volatile suspended solids per litre) in each BER and Control.

A synthetic medium resembling groundwater was used to conduct the electrochemical reactions which aimed to induce denitrification. The synthetic medium was designed to resemble a polluted groundwater well in Chile, according to information published by the General Water Directorate (Dirección General de Aguas, in Spanish) (Villablanca Espinoza, 2016). The culture medium comprised nitrate 37.6 ± 1.2 mg NO_3_^−^-N/L (166.5 ± 5.2 mg NO_3_^−^/L) and the following compounds in mg/L: 528 NaHCO_3_, 465 MgSO_4_·7H_2_O, 282 CaCl_2_ and 249 Na_2_SO_4_. The final measured conductivity of the medium reached around 3,500 μS/cm.

### 2.3 Analytical methods

Homogenous bulk samples were taken from all reactors daily until nitrate and nitrite concentrations were stable. Then, these were centrifuged at 12,000 rcf for 15 min for analysis. The centrifugation pellets were stored at −20°C to further extract DNA (See Section 2.4), and the supernatant was filtered under 0.22 µm using syringe filters model FN2522 (Zhejiang Aijiren Technology, Inc., China) to obtain the soluble fraction. Concentrations of nitrate, nitrite and sulfate were monitored using an ion chromatograph model 930 Compact IC (Metrohm, Switzerland), coupled with a conductivity detector using a Metrosep A Supp 5–150/2.0 column (Metrohm, Switzerland). To ensure the ionization of the targeted analytes a solution of 1 mmol/L and 3.2 mmol/L of NaHCO_3_ and Na_2_CO_3_ respectively, were used as mobile phase, at a flow rate of 0.7 mL/min. Total ammoniacal nitrogen (TAN) was measured using the Hach kit nitrogen, ammonia reagent set (Hach, United States) using a portable colorimeter DR 900 from the same brand. Total suspended solids were measured according to Standard Methods protocols (Rice et al., 2017), using a glass fibre filter with a nominal pore size of 0.4 µm (MACHEREY-NAGEL, Germany).

### 2.4 Bacterial community analysis

DNA samples were taken from the inoculum (Inoc) and from reactors during denitrification time at 1V (1V_1_-d1, 1V_1_-d2, 1V_2_-d1 and 1V_2_-d2), 2V (2V-d6, 2V-d7 and 2V-d14) and Control (C-d9 and C-d15). The samples were selected according to the denitrification dynamics observed under the conditions studied. DNA was extracted using the DNeasy PowerSoil Pro Kit (Qiagen, Germany) according to the manufacturer’s instructions. Quality and quantity of the extracted DNA were analysed using a Take3 microvolume plate (BioTek Instruments, United States) and measured with a spectrophotometer model Epoch (BioTek Instruments, United States) and a fluorometer model Fluo-100 (Hangzhou Allsheng Instruments Co., Ltd., China), respectively. The V4 variable region of the 16S rRNA gene was amplified using 515F/806R primers in a single-step 30-cycles PCR reaction, utilizing a HotStarTaq Plus Master Mix Kit (Qiagen, United States). The PCR conditions are described as follows: 95°C for 5 min, followed by 30 cycles of 95°C for 30 s, 53°C for 40 s and 72°C for 1 min, after which a final elongation step at 72°C for 10 min was performed. To confirm the success of the amplification, the PCR products were analysed in 2% agarose gel and the relative intensity of the bands formed was determined. The amplified samples were multiplexed using unique dual indices and were pooled together in equal proportions, based on their molecular weight and DNA concentrations. Pooled samples were purified using calibrated AMPure XP beads (Beckman Coulter, Inc., United States). The sequencing was performed at Mr DNA laboratory (www.mrdnalab.com, Shallowater, TX, United States) in a MiSeq (Illumina Inc., United States) following the manufacturer’s guidelines. Sequenced data were processed using Mr DNA lab analysis pipeline (MR DNA, Shallowater, TX, United States). Clustering of the remaining sequences into OTUs was executed based on a divergence threshold of 3%. For taxonomic classification of the final OTUs, a curated database sourced from GreenGenes, RDPII, and NCBI was employed, utilizing BLASTn (DeSantis et al., 2006) (http://rdp.cme.msu.edu; www.ncbi.nlm.nih.gov). Sequence data were uploaded into NCBI GenBank database submission number PP819758–PP820317.

### 2.5 Data analysis and statistical tools

Shannon diversity index (H) was calculated to compare the variation in the selected communities according to the applied voltage. In addition, principal component analysis (PCA) was performed from variance-covariance matrix based on genus distribution of the bacterial communities during the bioelectrochemical processes. Indicator species analysis (IndVar) was also calculated to determine the characteristic genera in the bacterial communities selected under the conditions studied. In addition, a similarity percentage test (SIMPER) was performed to determine which genera of the bacterial community contributed to the differences between the conditions studied. Besides, SIMPER was calculated using Bray-Curtis distance, in which only genera with a contribution to dissimilarity higher than 1.0% are shown. All statistical analyses were conducted using the PAST (PAleontological STatistics) software v4.16c (website https://www.nhm.uio.no/english/research/resources/past/) (Hammer and Harper, 2001).

## 3 Results

### 3.1 Reactor performance during nitrate removal

**FIGURE 1:**
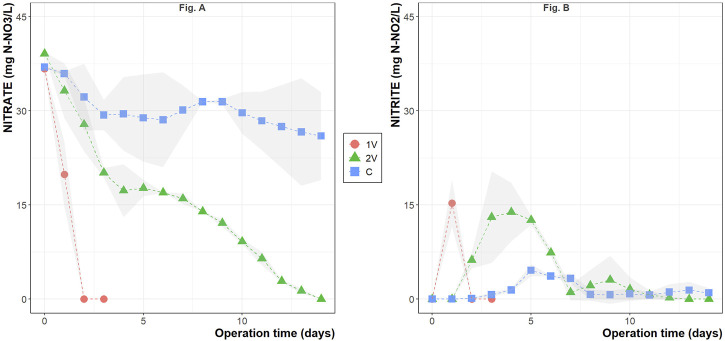
Nitrate **(A)** and nitrite **(B)** concentrations during the conducted electro-assisted autotrophic denitrification experiments. The plotted values represent the mean of the duplicates, and the gray-shaded area the standard deviation among the data.

Figure 1A shows the nitrate reduction during the operation time of the two BER during autotrophic denitrification in nitrate and sulfate-rich synthetic groundwater. Nitrate reduction was observed in the first 48 h and 14 days of operation when 1V and 2V were applied, respectively. The nitrate removal rate of 1V was considerably higher than 2V, averaging 18.8 ± 0.6 mg NO_3_^−^-N/Ld (11.28 ± 0.72 g NO_3_^−^-N/m^2^·d) compared to 2.7 ± 0.1 mg NO_3_^−^-N/Ld (1.62 ± 0.06 g NO_3_^−^-N/m^2^·d), respectively. During the Control experiments, *i.e.*, without electrodes, between 13.8% ± 2.0% and 43.6% ± 0.9% of nitrate was reduced during the experiment. In addition, no chemical reduction of nitrate was detected in the abiotic controls. In addition, no electrochemical reduction of nitrate was detected in the abiotic controls at 1V and 2V (data available in the Supplementary Materials).

Figure 1B shows that the maximum nitrite concentrations reached 15.3 ± 3.8 mg NO_2_^−^-N/L at 1V, 13.9 ± 4.7 mg NO_2_^−^-N/L at 2V and 4.6 ± 0.9 mg NO_2_^−^-N/L in the Control in the days 1, 4 and 5, respectively. In all cases, nitrite was rapidly converted and reached zero at the end of the operation.

The pH in the reactors was monitored during the whole operation time and varied from 8.2 ± 0.2 at day 0–7.6 ± 0.2 at day 14 in all the studied cases. Sulfate was also monitored, and despite the high sulfate content in the synthetic groundwater, no sulfate reduction was observed in any reactor (data available in the Supplementary Materials). TAN production was also not detected in any of the studied reactors. As for the electro-assisted operation of the experiments, a current flow smaller than the detection threshold of 1 mA was observed.

### 3.2 Bacterial community structure

DNA samples were taken from the inoculum (Inoc) and from reactors during denitrification time at 1V (1V_1_-d1, 1V_1_-d2, 1V_2_-d1 and 1V_2_-d2), 2V (2V-d6, 2V-d7 and 2V-d14) and Control (C-d9 and C-d15). A total of 890 operational taxonomic units (OTUs) were found after MiSeq sequencing in all samples. The Shannon diversity index (H) in the inoculum reached 3.27, and a strong decrease in diversity of 21% ± 4% (H = 2.59 ± 0.12) was observed when 1V was applied. In contrast, when 2V was applied, diversity increased from day 6 (H = 3.99) to day 14 (H = 4.32) between 21.9% and 32.1%, respectively. While in the Control, a slight decrease in diversity of 5.9% was observed on day 9 (H = 3.08), but by day 15 (H = 3.48) it increased by 6.3% (more details in the Supplementary Materials).

**FIGURE 2:**
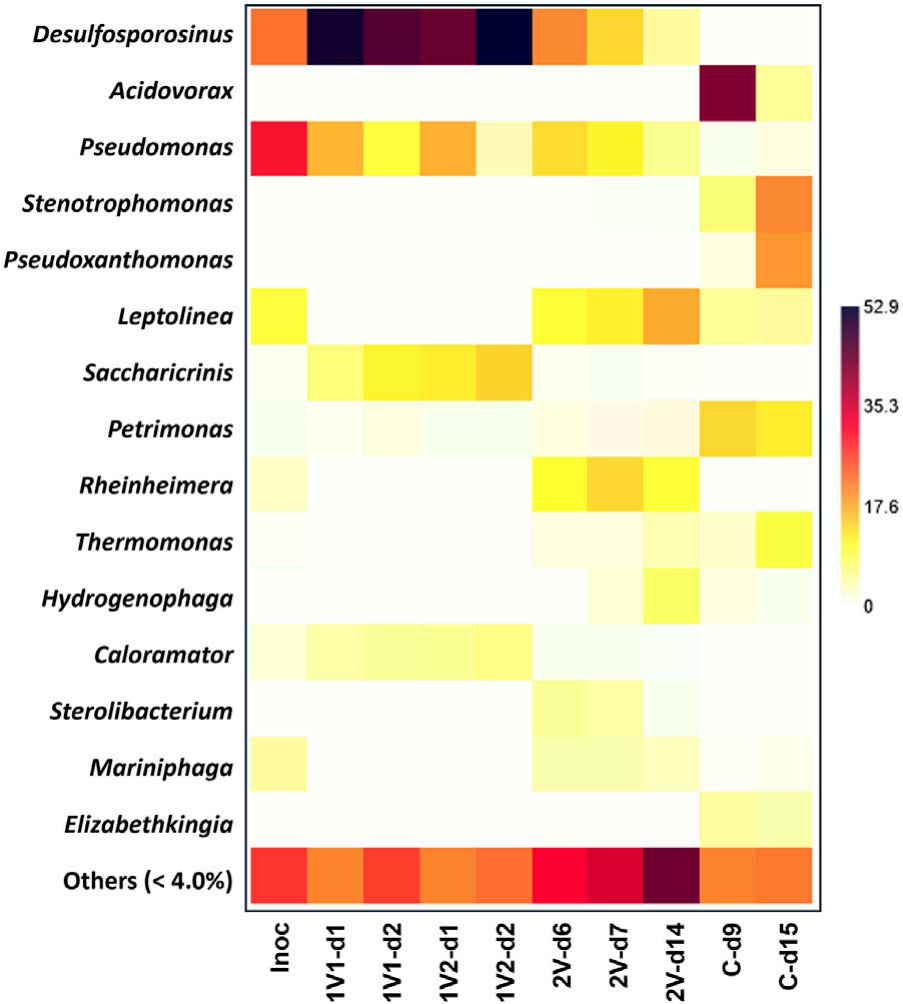
Heatmap of the bacterial community structure based on relative abundance at genus taxonomic level according to the condition studied.

Figure 2 shows a heatmap including the relative abundance of bacterial genera in each bacterial community, according to the condition studied. The bacterial community present in Inoc was dominated by the genus *Pseudomonas* (29.9%), *Desulfosporosinus* (22.1%), *Leptolinea* (9.6%) and *Mariniphaga* (4.8%). When 1V was applied, three genera were significantly enriched with respect to Inoc: *Desulfosporosinus*, *Saccharicrinis* and *Caloramator* representing 48.4% ± 4.2%, 11.0% ± 3.1% and 5.3% ± 0.7% of the bacterial community, respectively. Additionally, the genus *Pseudomonas* decreased its relevance in the bacterial community, reaching 16.6% ± 0.3% during the first 24 h of operation and to 6.6% ± 4.3% when at 48 h of operation. When 2V was applied, substantial differences in the bacterial community between days 6 and 7 of operation were not observed, and on average, the dominant genera were *Desulfosporosinus* (17.0% ± 4.6%), *Pseudomonas* (12.2% ± 1.5%), *Rheinheimera* (12.0% ± 2.1%), *Leptolinea* (11.0% ± 1.1%), *Sterolibacterium* (4.8% ± 0.5%) and *Mariniphaga* (4.1% ± 0.03%). However, on day 14 at the end of the operation, the bacterial community was dominated by *Leptolinea* (17.4%), *Rheinheimera* (10.0%), *Hydrogenophaga* (7.8%), *Pseudomonas* (5.6%) and *Desulfosporosinus* (4.8%). When it comes to the control samples, at 9 days of operation, the bacterial community was dominated by *Acidovorax* (42.1%), *Petrimonas* (13.8%), *Stenotrophomonas* (7.0%), *Leptolinea* (5.1%) and *Elizabethkingia* (4.6%). However, on day 15 at the end of the operation, the bacterial community was dominated by *Stenotrophomonas* (20.3%), *Pseudoxanthomonas* (19.0%), *Petrimonas* (11.9%), *Thermomonas* (9.5%), *Acidovorax* (5.1%), *Leptolinea* (5.0%) and *Elizabethkingia* (4.2%).

### 3.3 Relationship between the voltage applied and bacterial community selection

**FIGURE 3:**
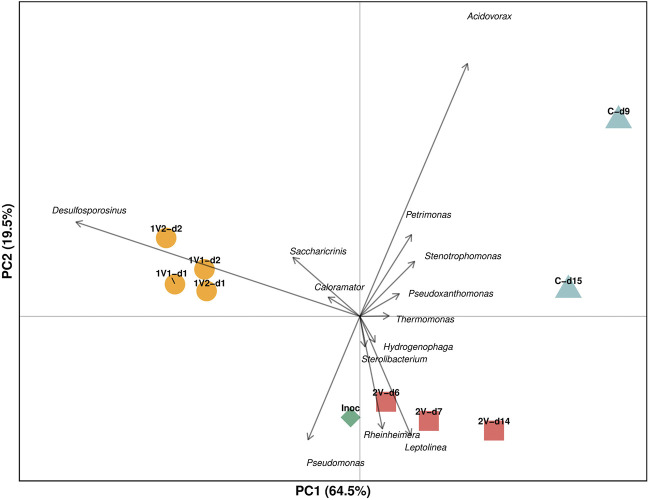
PCA Biplot based on bacterial community distribution at genus level according to the conditions studied.

Figure 3 shows a biplot of the PCA performed based on genus distribution of the bacterial communities during the denitrification process. About 85% of the variance was explained by the two first components, evidencing high reliability in this data analysis. PCA allows better visualization of the bacterial community dynamics from the Inoc to operation at different applied voltages, revealing characteristic bacterial genus groups for each condition. Thus, the genera *Desulfosporosinus*, *Saccharicrinis* and *Caloramator* are related to the 1V operation, while *Rheinheimera*, *Leptolinea*, *Hydrogenophaga* and *Sterolibacterium* are related to 2V operation. The Control communities, although located in the same quadrant, show greater dispersion along *y*-axis, evidencing a variation of the bacterial community between days 9 and 15. Despite these differences, the genera *Acidovorax*, *Stenotrophomonas* and *Pseudoxanthomonas* are mainly correlated with the Control. Since PC1 explains 64.5% of the total variability in the data, the separation along the *x*-axis in the PCA is more relevant than along the *y*-axis. In this context, the Control is positioned on the far right, while the 1V operation is on the far left, reflecting the greater distance between the two. The 2V operation, on the other hand, appears close to the Inoc, indicating that it generated minimal changes in the bacterial community composition with respect to the Inoc, while the 1V treatment produced the most pronounced modifications on the community, positioning that community in a direction opposite to that of the Control.

An Indicator Species Analysis (IndVar) was performed to determine the characteristic genera in the bacterial communities selected under conditions studied, as shown in Table 1. For this analysis, the samples were grouped as 1V, 2V, Control and Inoc. The characteristic genera of the selected community when 1V was applied were *Desulfosporosinus*, *Saccharicrinis* and *Caloramator* with an indicator value of 58.0% (*p-value* = 0.0048), 90.3% (*p-value* = 0.0022) and 68.4% (*p-value* = 0.0044), respectively. For the selected bacterial community when 2V was applied, it is observed that *Rheinheimera*, *Hydrogenophaga* and *Sterolibacterium*, are characteristic of this condition with an indicator value of 80.6% (*p-value* = 0.0073), 76.8% (*p-value* = 0.046) and 99.2% (*p-value* = 0.0057), respectively. In the Control reactor, a higher number of species with a significant indicator value were found, including *Acidovorax* (98.9%, *p-value* = 0.013), *Stenotrophomonas* (97.4%, *p-value* = 0.013), *Pseudoxanthomonas* (98.3%, *p-value* = 0.013), *Petrimonas* (80.8%, *p-value* = 0.0072), *Thermomonas* (69.5%, *p-value* = 0.046) and *Elizabethkingia* (100%, *p-value* = 0.021). While in Inoc, the genus *Pseudomonas* was the only one significantly characteristic with an indicator value of 56.9% (*p-value* = 0.0018).

**TABLE 1:** IndVar based on bacterial community distribution at genus level and grouped according to voltage applied. Statistical significance was assessed considering a *p-value ≤ 0.05* (*) and *≤0.01* (**).

Genus	1V	2V	C	Inoc
*Desulfosporosinus*	58.0**	15.5	0.1	26.5
*Acidovorax*	0.2	0.5	98.9*	0.3
*Pseudomonas*	22.1	19.0	2.0	56.9**
*Stenotrophomonas*	0.2	1.9	97.4*	0.4
*Pseudoxanthomonas*	0.1	1.5	98.3*	0.2
*Leptolinea*	0.2	47.3	18.2	34.4
*Saccharicrinis*	90.3**	4.3	0.5	4.9
*Petrimonas*	5.8	8.4	80.8**	5.1
*Rheinheimera*	0.1	80.6**	0.0	19.3
*Thermomonas*	0.4	25.3	69.5*	4.7
*Hydrogenophaga*	0.0	76.8*	23.1	0.0
*Caloramator*	68.4**	8.4	0.2	22.9
*Sterolibacterium*	0.0	99.2**	0.8	0.0
*Mariniphaga*	0.3	40.4	7.6	51.8
*Elizabethkingia*	0.0	0.0	100*	0.0

In addition to the formerly described analysis, a SIMPER test was performed to better understand which bacterial genera are significantly responsible for the differences observed between the conditions studied (Table 2). A comparison between the electro-assisted reactors (1V + 2V) and Control revealed a dissimilarity of 87.5%, primarily attributed to *Desulfosporosinus* (19%), *Acidovorax* (13.5%), *Stenotrophomonas* (7.7%), *Petrimonas* (6.7%), *Pseudoxanthomonas* (5.8%) and *Pseudomonas* (5.7%). Among these, *Desulfosporosinus* and *Pseudomonas* were more abundant in the electro-assisted reactors, while *Acidovorax*, *Stenotrophomonas*, *Petrimonas* and *Pseudoxanthomonas* were more abundant in the Control. Comparing between electro-assisted reactors (1V vs 2V), a dissimilarity of 71.5% was observed, mainly by the abundance of *Desulfosporosinus* (24.9%), *Leptolinea* (9.2%), *Rheinheimera* (7.9%), *Saccharicrinis* (7.4%) and *Pseudomonas* (4.0%). *Desulfosporosinus, Saccharicrinis* and *Pseudomonas* were more abundant in 1V reactors, while *Leptolinea* and *Rheinheimera* in 2V reactors.

**TABLE 2:** SIMPER analysis performed to compare the genus bacterial composition of electro-assisted reactors.

Genus	1V and 2V (%)		(1V+2V) and control (%)	
	Dissimilarity Contrib^a^ ^,^ ^b^	Total	Dissimilarity Contrib^a^ ^,^ ^b^	Total^c^
*Desulfosporosinus*	**24.9**	24.9	**19.0**	19.0
*Acidovorax*	0.1	24.9	**13.5**	32.5
*Leptolinea*	**9.2**	34.1	3.7	36.2
*Rheinheimera*	**7.9**	42.0	2.8	39.0
*Stenotrophomonas*	0.2	42.2	**7.7**	46.7
*Saccharicrinis*	**7.4**	49.6	3.6	50.3
*Petrimonas*	0.3	49.9	**6.7**	57.1
*Pseudoxanthomonas*	0.1	50.0	**5.8**	62.9
*Pseudomonas*	**4.0**	54.0	**5.7**	68.6
*Caloramator*	3.2	57.2	1.9	70.4
*Thermomonas*	1.5	58.7	3.0	73.4
*Mariniphaga*	2.6	61.4	1.0	74.4
*Elizabethkingia*	0.0	61.4	2.5	76.9
*Sterolibacterium*	2.5	63.8	0.9	77.8
*Hydrogenophaga*	2.3	66.2	1.0	78.9
*Anaerobaculum*	2.1	68.2	1.0	79.8
*Moorella*	1.8	70.0	0.8	80.7
*Simplicispira*	1.6	71.7	0.6	81.2
*Thermoanaerobacter*	1.3	72.9	0.8	82.1
*Spongiimonas*	1.2	74.2	0.6	82.7
*Comamonas*	1.2	75.3	0.4	83.1
*Imtechium*	1.1	76.5	0.4	83.5
*Fluviicola*	1.1	77.6	0.4	83.9
*Thiobacillus*	1.1	**78.7**	0.6	**84.5**

^a^ Dissimilarity contrib.: correspond to percentage that each genus is contributing to dissimilarity between the groups compared.

^b^ Only genera that contribute ≥1.0%, in at least one sample, to the dissimilarity are included in the table.

^c^ Total: correspond to accumulative contribution of each genus to dissimilarity percentage.

## 4 Discussion

### 4.1 Electro-assisted autotrophic denitrification performances

When working with BER, researchers generally seek to promote the formation of an electroactive biofilm during 2–3 weeks of acclimation, which ends when a significant increase in electron flow is observed (Patil et al., 2010; Mier et al., 2021; Hackbarth et al., 2023). Interestingly, in some systems working in mode “Electro-fermentation” there is no electroactive biofilm formation. In these cases, the electron fluxes are very small, in the order of µA, but significant changes in microbial communities and fermentation products are observed (Toledo-Alarcón et al., 2019; 2021; Cardeña et al., 2024). Our results show an increase of the autotrophic denitrification rate associated with changes in the bacterial diversity. Specifically, BERs operated at 1V had a denitrification rate 40% higher than the maximum reported (8.19 ± 0.97 g NO_3_^−^-N/m^2^·d) (Feng et al., 2024), while the BER operated at 2V is comparable with typical removal rates ranging from 0.23–3.67 g NO_3_^−^-N/m^2^·d, including single and double chamber BERs (Pous et al., 2016; Liu et al., 2019; Vijay et al., 2019; Lin et al., 2021; Zhao et al., 2023). Denitrification is fully associated with bacterial activity as no electrochemical reduction of nitrate was observed in the abiotic controls at 1V and 2V.

Sulfate and nitrate were added to synthetic groundwater to mimic the composition of a real one. Both molecules are known to function as final electrons acceptors during anoxic growth of some bacteria. However, as shown in Equation 1 and Equation 2, nitrate is expected to be consumed preferentially over sulfate, as the nitrate reduction pathway provides more free energy per electron to the bacteria (Campos et al., 2019). Nitrate reduction was conducted via denitrification, as the TAN concentrations measured during the experimental time were zero, showing that dissimilarity reduction of nitrate to ammonia did not occur. The dissimilatory reduction of nitrate to ammonia tends to occur at high C/N ratios and with organic carbon sources (Utting et al., 2011; Chutivisut et al., 2018), conditions that were not present in our experiments.

### 4.2 Direct electron consumption in the selected denitrifying bacterial community at 1V

Selected bacterial community in the BERs operated at 1V was largely dominated by the genus *Desulfosporosinus*, a group of strict anaerobic and well-known sulfate-reducing bacteria (Pester et al., 2012). However, species such as *Desulfosporosinus acididurans* have been reported to be nitrate-reducing (Sánchez-Andrea et al., 2015). As for the electron source, electroactive growth by consuming electrons from a cathode has been reported in *Desulfosporosinus orientis*. Although the mechanism of interaction with the electrode remains unknown, both biological and abiotic H_2_ production as an electron mediator have been hypothesised (deCamposRodrigues and Rosenbaum, 2014; Agostino and Rosenbaum, 2018; Agostino et al., 2020). Besides, enrichment of the genus *Desulfosporosinus* from a mixed community has been reported during the simultaneous reduction of sulfate and nitrite in electro-assisted reactors (Chai et al., 2020).

The genus *Caloramator* was also enriched, which is composed of strict anaerobic and fermentative species that have been reported in reactors producing H_2_ under thermophilic conditions (Seyfried et al., 2002; Rubiano-Labrador et al., 2013). Interestingly, this genus has recently been proposed as electroactive, as it has been reported to play a key role in the electrons transport using conductive materials and in the production of electricity in microbial fuel cells. (Fu et al., 2013; Yan et al., 2017). However, its mechanism of EET is so far unknown.

Another characteristic genus selected in BER at 1V was *Saccharicrinis*, which is composed of facultative anaerobic species that have been reported in reactors associated with heterotrophic denitrification (Sposob et al., 2020; Han et al., 2021) but there is no evidence of species that can perform autotrophic denitrification (Liu et al., 2014; Yang et al., 2014). There is evidence that the application of a voltage could generate important changes on the surface of the bacterial membrane, causing even cellular decay (Krishnamurthi et al., 2020). This decay could make organic molecules available to the bacteria to use them as substrates during heterotrophic denitrification.

Considering the significant differences in the rate of nitrate consumption between Control and 1V, together with the metabolic characteristics of the selected bacterial community, we hypothesize that denitrification occurred primarily through direct consumption of electrons from the cathode.

### 4.3 Water electrolysis at 2V and its effect on denitrifying bacterial community selection

Unlike the experiments performed at 1V, the experiments at 2V were mainly driven by water electrolysis products. This is expected to occur from 1.23 V under standard conditions (Lamy and Millet, 2020). Water electrolysis leads to O_2_ and H_2_ formation, which can be used as electron acceptors and donors, respectively. Despite the low current observed in our present work (<1 µA), H_2_ and O_2_ formation could drive changes in the bacterial community, even though H_2_ and O_2_ were not detected in the headspace nor in the reactor. This is probably because the consumption rate of these gases was faster than the production rate and saturation was not reached in the liquid. The presence of dissolved gases in the bulk without reaching saturation to be desorbed to the headspace is a common phenomenon in microbial systems; in these cases, the measurement of oxidation-reduction potential is a more useful strategy to monitor the processes (Krayzelova et al., 2015; Illi et al., 2021; Fu et al., 2023).

Consequently, the electrons used during denitrification at 2V were supplied from H_2_ oxidation. However, electroactive denitrification using electrons transferred directly from the cathode (in the same way as at 1V) could also occur due to the abundance of the *Desulfosporosinus* genus, especially early in the BER operation. Electrolysis-based H_2_ occurrence was supported by the enrichment of the genus *Hydrogenophaga*. Members of the genus *Hydrogenophaga* are aerobic or facultative anaerobes (Banerjee et al., 2021) which can oxidize H_2_ as an energy source reducing CO_2_ as carbon source (Blohm et al., 2022; Thorat et al., 2022). In addition, there is evidence that members of this genus can completely reduce nitrate to dinitrogen (Banerjee et al., 2021). To the authors’ knowledge, *Hydrogenophaga* has not been described as an electroactive bacterium although its enrichment has already been reported in denitrifying BER (Zhao et al., 2017; Peng et al., 2018; Yao et al., 2022). Consequently, in our assays we attribute the increase in their relative abundance to nitrate reduction using H_2_ as an energy source, as previously described by (Pous et al., 2022).

Besides *Hydrogenophaga,* the genera *Rheinheimera*, *Leptolinea, and Sterolibacterium* predominated the experiments at 2V. *Leptolinea tardivitalis* is the only known species of genus *Leptolinea,* which is a strictly anaerobic, heterotrophic bacterium that cannot utilize nitrate as electron acceptor (Yamada et al., 2006). *Rheinheimera* genus is a chemoheterotrophic bacterium that can grow in aerobic and facultative-anaerobic conditions. This genus can also reduce nitrate to nitrite in some aerobic species such as *R. aestuarii H29T, R. pacifica CCUG 46544T, R. baltica DSM 14885T* (Baek and Jeon, 2015) and some facultative anaerobic (*e.g., Rheinheimera texasensis A62-14BT,* and *R. perlucida BA131T*) (Merchant et al., 2007). *Rheinmera* genus has been associated with nitrogen removal on the cathode in anoxic environments (Xie et al., 2016) and has been reported growing in the planktonic biomass near the cathode, which means that it relies on mediators such as H_2_, flavins, quinones and phenazines (Lin et al., 2020).

Within the *Sterolibacterium* genus only one species is known, *Sterolibacterium denitrificans*, which is a facultative anaerobic bacterium (Chiang et al., 2008), capable of reducing nitrate using cholesterol as an electron donor (Tarlera and Denner, 2003). Since cholesterol and other sterols persist in an anaerobic digestate (Weckerle et al., 2023), they can be utilized by a specific microbial community as electron donors. In addition, *Sterolibacterium* have been reported to increase their relative abundance in O_2_-limited and nitrate-rich environments (Chen et al., 2024). Although the *Sterolibacterium* genus is not confirmed to be electroactive, it has been reported growing in the anode of an electrochemical biofilter for the treatment of municipal wastewater (Yang et al., 2018) and in the anode of a microbial fuel cell treating cattle manure slurry (Xie et al., 2017).

In addition to the denitrification reactions that occurred at 2V, oxidation reactions may have also occurred because of O_2_ presence due to electrolysis. Consequently, O_2_ may have competed with nitrate and nitrite as final electron acceptors. The lower nitrate consumption rate at 2V compared with the experiment performed at 1V could result from the competition of O_2_ and nitrate (Ortega-Martínez et al., 2024).

### 4.4 Endogenous respiration in the control bacterial community

Bacterial community selected in the Control experiments included mainly the genus *Acidovorax* during the first days of operation. This genus has been widely reported to be dominant in heterotrophic, autotrophic and mixotrophic denitrifying systems (Jiang et al., 2020; Tian and Wang, 2021; Ren et al., 2022; Bai et al., 2023; Zhang et al., 2024). Some species known to perform complete heterotrophic denitrification include *A. delafieldii* and *A. temperans*. *A. avenae* has been reported to be associated with autotrophic denitrification (Fernandez et al., 2009), while other species such as *A. facilis* and *A. konjaci* can reduce nitrate only to nitrite (Willems and Gillis, 2015). The genus *Petrimonas*, to which bacterial species capable of using nitrate as an electron acceptor belong, was also relevant. This genus has been reported to be dominant in denitrifying systems (Whitman et al., 2015; Li et al., 2016; Hu et al., 2023), while *Stenotrophomonas* and *Pseudoxanthomonas* are genera of Gram-negative bacteria of the family Xanthomonadaceae that have been reported in denitrifying reactors, but commonly as minority species (Mahto and Das, 2022; Bai et al., 2023; Luan et al., 2023; Zhang et al., 2023).

Endogenous respiration is a common phenomenon that occurs when, in the absence of external substrates, the hydrolytic enzymes present in the anaerobic biomass cause lysis of the microorganisms (Van Loosdrecht and Henze, 1999). Furthermore, it is expected that the endogenous respiration rate increased when the biomass was exposed to anoxic conditions since the nitrate-using bacteria tend to have a higher endogenous respiration rate compared to anaerobes (Rieger et al., 2001). Moreover, there is evidence that the addition of external electron acceptors, such as nitrate, during anaerobic digestion can increase the relative abundance of fermentative bacteria, which are responsible for the production and excretion of hydrolytic enzymes (Nguyen and Khanal, 2018; Nguyen et al., 2019). Consequently, endogenous respiration could explain the decrease in nitrate concentration observed in the Control reactors, when using organic autolysis as an organic matter source for denitrification. The results show that this requires about 15–45 mg COD/L, which obtained from endogenous respiration is at most 3.0% of the COD contained in the inoculated biomass.

## 5 Conclusion

Our results demonstrate that the cathode can effectively serve as an electron donor for nitrate reduction in BER, with the applied voltage being a key factor influencing efficiency and microbial community dynamics. The highest nitrate removal rate was obtained at 1V, associated with the enrichment of the electroactive genus *Desulfosporosinus*, highlighting the potential of low-voltage operation to enhance denitrification without biofilm formation. In contrast, at 2V, O_2_ produced via water electrolysis competed with nitrate as electron acceptor, reducing the denitrification rate. The enrichment of *Hydrogenophaga* at 2V suggests that H_2_ generated by electrolysis contributed to nitrate reduction at this condition. These finding underscore the importance of optimizing the voltage applied for each reactor design to enhance nitrate removal efficiency. Our work represents a significant contribution to the scaling up of BER and their application in groundwater remediation, utilizing a single-chamber, membrane-free configuration, and a conventional power source instead of a potentiostat.

## Data Availability

The datasets associated with the bacterial community sequencing presented in this study can be found in online repositories. The names of the repository/repositories and accession number(s) can be found below: https://www.ncbi.nlm.nih.gov/genbank/, PP819758–PP820317.
