# Supplementary material for: Groundwater denitrification using electro-assisted autotrophic processes: exploring bacterial community dynamics in a single-chamber reactor
Source: Front Bioeng Biotechnol. 2025 Jan 22;13:1475589. doi: 10.3389/fbioe.2025.1475589 (PMC11794223; doi:10.3389/fbioe.2025.1475589)
Supplement: Supplementary file 1 [file Table1.docx]

Supplementary Material

# Shannon diversity index

Supplementary Table 1. Shannon diversity index

|  | **Shannon_H** |
| --- | --- |
| **1V1-d1** | 2.509 |
| **1V1-d2** | 2.771 |
| **1V2-d1** | 2.571 |
| **1V2-d2** | 2.522 |
| **2V-d6** | 3.986 |
| **2V-d7** | 4.159 |
| **2V-d14** | 4.319 |
| **C-d9** | 3.075 |
| **C-d15** | 3.475 |
| **Inoc** | 3.269 |

# Sulfate concentration data

Supplementary Table 2. Sulfate concentration (mg/L) data for the conditions studied. The average of the duplicates and the standard deviation (SD) of the data are shown.

| **Operation time (days)** | **1V** | | **2V** | | **Control** | |
| --- | --- | --- | --- | --- | --- | --- |
|  | Average | SD | Average | SD | Average | SD |
| 0 | 319.6 | 4.3 | 409.95 | 64.49 | 415.5 | 31.5 |
| 1 | 340.8 | 3.4 | 483.45 | 26.84 | 468.2 | 77.8 |
| 2 | 372.3 | 2.2 | 527.95 | 139.36 | 446.0 | 153.6 |
| 3 | 375.8 | 17.5 | 474.49 | 113.33 | 491.9 | 113.8 |
| 4 | - | - | 459.56 | 141.80 | 571.2 | 121.1 |
| 5 | - | - | 532.77 | 45.60 | 596.4 | 51.9 |
| 6 | - | - | 535.49 | 45.44 | 628.9 | 7.0 |
| 7 | - | - | 574.03 | 5.39 | 610.4 | 35.1 |
| 8 | - | - | 538.33 | 48.77 | 622.9 | 24.6 |
| 9 | - | - | 542.44 | 46.64 | 587.2 | 29.4 |
| 10 | - | - | 591.37 | 107.90 | 622.6 | 26.1 |
| 11 | - | - | 547.87 | 41.10 | 586.5 | 4.8 |
| 12 | - | - | 549.81 | 38.54 | 581.0 | 18.2 |
| 13 | - | - | 551.74 | 35.98 | 608.9 | 6.2 |
| 14 | - | - | 562.05 | 21.58 | 625.7 | 14.8 |

# Relative abundance of the bacterial community

Supplementary Table 3. Relative abundance (%) of the bacterial community at genus level. Only genus with a relative abundance greater than 1.0% are included.

| **Genus** | **1V1-d1** | **1V1-d2** | **1V2-d1** | **1V2-d2** | **2V-d6** | **2V-d7** | **2V-d14** | **C-d9** | **C-d15** | **Inoc** |
| --- | --- | --- | --- | --- | --- | --- | --- | --- | --- | --- |
| *Desulfosporosinus* | 51.0 | 45.8 | 44.0 | 52.9 | 20.2 | 13.7 | 4.8 | 0.1 | 0.1 | 22.1 |
| *Acidovorax* | 0.0 | 0.1 | 0.1 | 0.0 | 0.1 | 0.1 | 0.1 | 42.1 | 5.1 | 0.1 |
| *Pseudomonas* | 16.4 | 9.7 | 16.9 | 3.6 | 13.3 | 11.1 | 5.6 | 0.8 | 1.3 | 29.9 |
| *Stenotrophomonas* | 0.1 | 0.0 | 0.0 | 0.0 | 0.2 | 0.3 | 0.3 | 7.0 | 20.3 | 0.1 |
| *Pseudoxanthomonas* | 0.0 | 0.0 | 0.0 | 0.0 | 0.1 | 0.1 | 0.2 | 1.5 | 19.0 | 0.0 |
| *Leptolinea* | 0.0 | 0.1 | 0.0 | 0.0 | 10.2 | 11.8 | 17.4 | 5.1 | 5.0 | 9.6 |
| *Saccharicrinis* | 6.7 | 11.3 | 11.9 | 14.1 | 0.6 | 0.7 | 0.4 | 0.0 | 0.1 | 0.6 |
| *Petrimonas* | 0.6 | 1.3 | 0.9 | 0.9 | 1.3 | 1.1 | 1.6 | 13.8 | 11.9 | 0.8 |
| *Rheinheimera* | 0.0 | 0.0 | 0.0 | 0.0 | 10.4 | 13.5 | 10.0 | 0.0 | 0.0 | 2.7 |
| *Thermomonas* | 0.1 | 0.0 | 0.0 | 0.0 | 1.4 | 1.4 | 3.8 | 2.5 | 9.5 | 0.4 |
| *Hydrogenophaga* | 0.0 | 0.0 | 0.0 | 0.0 | 0.2 | 2.0 | 7.8 | 1.3 | 0.8 | 0.0 |
| *Caloramator* | 4.4 | 5.2 | 5.4 | 6.1 | 0.9 | 0.8 | 0.3 | 0.0 | 0.0 | 1.8 |
| *Sterolibacterium* | 0.0 | 0.0 | 0.0 | 0.0 | 5.2 | 4.4 | 0.9 | 0.0 | 0.1 | 0.0 |
| *Mariniphaga* | 0.0 | 0.0 | 0.0 | 0.0 | 4.1 | 4.1 | 3.2 | 0.4 | 1.0 | 4.8 |
| *Elizabethkingia* | 0.0 | 0.0 | 0.0 | 0.0 | 0.0 | 0.0 | 0.0 | 4.6 | 4.2 | 0.0 |
| *Anaerobaculum* | 2.6 | 3.8 | 2.5 | 3.0 | 0.0 | 0.0 | 0.0 | 0.0 | 0.0 | 0.0 |
| *Simplicispira* | 0.0 | 0.0 | 0.1 | 0.0 | 1.4 | 1.9 | 3.7 | 0.1 | 0.1 | 0.3 |
| *Moorella* | 1.7 | 2.3 | 2.7 | 3.5 | 0.0 | 0.0 | 0.0 | 0.0 | 0.0 | 0.0 |
| *Thermoanaerobacter* | 2.8 | 2.1 | 2.8 | 1.4 | 0.6 | 0.5 | 0.2 | 0.0 | 0.0 | 1.0 |
| *Clostridium* | 1.8 | 2.4 | 1.4 | 1.9 | 0.4 | 0.5 | 0.8 | 0.3 | 0.2 | 0.5 |
| *Fluviicola* | 0.0 | 0.0 | 0.0 | 0.0 | 0.5 | 2.1 | 2.2 | 0.0 | 0.0 | 0.0 |
| *Spongiimonas* | 1.2 | 2.1 | 1.9 | 2.1 | 0.1 | 0.1 | 0.0 | 0.0 | 0.0 | 0.1 |
| *Lacibacter* | 0.0 | 0.0 | 0.0 | 0.0 | 0.0 | 0.0 | 2.1 | 0.0 | 0.0 | 0.0 |
| *Flavobacterium* | 0.0 | 0.0 | 0.0 | 0.0 | 0.9 | 1.4 | 0.5 | 2.0 | 0.6 | 0.1 |
| *Proteiniphilum* | 0.6 | 1.6 | 0.9 | 1.2 | 0.2 | 0.2 | 0.5 | 1.9 | 2.0 | 0.2 |
| *Marinomonas* | 2.0 | 1.4 | 1.9 | 0.7 | 0.9 | 0.6 | 0.2 | 0.0 | 0.0 | 1.6 |
| *Imtechium* | 0.0 | 0.0 | 0.0 | 0.0 | 1.9 | 2.0 | 1.1 | 0.0 | 0.1 | 0.2 |
| *Ochrobactrum* | 0.0 | 0.0 | 0.0 | 0.0 | 0.5 | 0.8 | 1.9 | 0.2 | 0.2 | 0.4 |
| *Thiobacillus* | 0.0 | 0.0 | 0.0 | 0.0 | 1.3 | 1.4 | 1.9 | 0.7 | 1.9 | 0.0 |
| *Leucobacter* | 0.0 | 0.0 | 0.0 | 0.0 | 0.9 | 0.8 | 1.9 | 0.9 | 1.1 | 1.0 |
| *Psychrobacter* | 0.2 | 1.8 | 0.1 | 0.4 | 0.0 | 0.0 | 0.0 | 0.0 | 0.0 | 0.0 |
| *Aquamicrobium* | 0.0 | 0.0 | 0.0 | 0.0 | 0.0 | 0.1 | 0.3 | 1.5 | 1.8 | 0.0 |
| *Comamonas* | 0.0 | 0.0 | 0.0 | 0.0 | 1.6 | 1.7 | 1.7 | 0.4 | 0.3 | 1.0 |
| *Parabacteroides* | 0.0 | 0.0 | 0.0 | 0.0 | 1.3 | 1.2 | 1.4 | 0.6 | 0.6 | 1.6 |
| *Hydrogenispora* | 0.1 | 0.0 | 0.0 | 0.0 | 0.0 | 0.0 | 0.0 | 0.0 | 0.0 | 1.5 |
| *Ottowia* | 0.1 | 0.0 | 0.0 | 0.0 | 1.1 | 0.9 | 0.9 | 1.4 | 1.5 | 0.8 |
| *Proteiniclasticum* | 0.0 | 0.0 | 0.0 | 0.0 | 0.8 | 0.9 | 1.4 | 1.0 | 0.6 | 0.7 |
| *Lachnobacterium* | 0.8 | 1.3 | 0.7 | 1.0 | 0.7 | 0.7 | 0.7 | 0.2 | 0.3 | 0.4 |
| *Thermovirga* | 0.0 | 0.0 | 0.0 | 0.0 | 0.9 | 1.0 | 0.6 | 0.0 | 0.1 | 1.3 |
| *Candidatus Atelocyanobacterium* | 0.0 | 0.0 | 0.0 | 0.0 | 0.5 | 0.5 | 1.2 | 0.1 | 0.3 | 0.6 |
| *Thauera* | 0.0 | 0.0 | 0.0 | 0.0 | 0.5 | 0.6 | 1.1 | 0.1 | 0.2 | 0.5 |
| *Ornatilinea* | 0.0 | 0.0 | 0.0 | 0.0 | 0.6 | 0.6 | 1.1 | 0.2 | 0.2 | 0.9 |
| *Alcanivorax* | 1.1 | 0.6 | 1.0 | 0.5 | 0.5 | 0.4 | 0.2 | 0.0 | 0.0 | 0.6 |
| *Acinetobacter* | 0.0 | 0.0 | 0.0 | 0.0 | 0.2 | 0.3 | 0.1 | 1.0 | 0.5 | 0.1 |
| *Polynucleobacter* | 0.0 | 0.0 | 0.0 | 0.0 | 0.0 | 0.0 | 0.0 | 1.0 | 0.3 | 0.0 |
| *Janthinobacterium* | 0.0 | 0.0 | 0.0 | 1.0 | 0.0 | 0.0 | 0.0 | 0.0 | 0.0 | 0.0 |
| Others (<1.0%) | 5.5 | 7.0 | 4.7 | 5.6 | 13.2 | 13.5 | 15.7 | 7.0 | 8.8 | 11.4 |

# Nitrate Abiotic control at 1V and 2V

Supplementary Table 4. Nitrate concentration (mg N-NO_3_^-^/L) data for the conditions studied. The average of the duplicates and the standard deviation (SD) of the data are shown.

| **Operation time**  **(days)** | **1V** | | **2V** | |
| --- | --- | --- | --- | --- |
|  | Average | SD | Average | SD |
| 0 | 46.2 | 0.8 | 47.4 | 0.6 |
| 2 | 46.2 | 1.9 | 47.7 | 2.1 |
